# Acquisition of *Pseudomonas aeruginosa* and its resistance phenotypes in critically ill medical patients: role of colonization pressure and antibiotic exposure

**DOI:** 10.1186/s13054-015-0916-7

**Published:** 2015-05-04

**Authors:** Nazaret Cobos-Trigueros, Mar Solé, Pedro Castro, Jorge Luis Torres, Cristina Hernández, Mariano Rinaudo, Sara Fernández, Álex Soriano, José María Nicolás, Josep Mensa, Jordi Vila, José Antonio Martínez

**Affiliations:** Department of Infectious Diseases, Hospital Clínic, Institut d’Investigacions Biomèdiques August Pi i Sunyer (IDIBAPS), University of Barcelona, Barcelona, Spain; ISGlobal, Barcelona Center for International Health Research (CRESIB), Hospital Clínic, University of Barcelona, Barcelona, Spain; Medical Intensive Care Unit, Hospital Clínic, Institut d’Investigacions Biomèdiques August Pi i Sunyer (IDIBAPS), University of Barcelona, Barcelona, Spain; Department of Clinical Microbiology, Hospital Clinic, School of Medicine, University of Barcelona, Barcelona, Spain; Department of Internal Medicine, University Hospital of Salamanca, Institute of Biomedical Research of Salamanca (IBSAL), Salamanca, Spain

## Abstract

**Introduction:**

The objective of this work was to investigate the risk factors for the acquisition of *Pseudomonas aeruginosa* and its resistance phenotypes in critically ill patients, taking into account colonization pressure.

**Methods:**

We conducted a prospective cohort study in an 8-bed medical intensive care unit during a 35-month period. Nasopharyngeal and rectal swabs and respiratory secretions were obtained within 48 hours of admission and thrice weekly thereafter. During the study, a policy of consecutive mixing and cycling periods of three classes of antipseudomonal antibiotics was followed in the unit.

**Results:**

Of 850 patients admitted for ≥3 days, 751 (88.3%) received an antibiotic, 562 of which (66.1%) were antipseudomonal antibiotics. A total of 68 patients (8%) carried *P. aeruginosa* upon admission, and among the remaining 782, 104 (13%) acquired at least one strain of *P. aeruginosa* during their stay. Multivariate analysis selected shock (odds ratio (OR) =2.1; 95% confidence interval (CI), 1.2 to 3.7), intubation (OR =3.6; 95% CI, 1.7 to 7.5), enteral nutrition (OR =3.6; 95% CI, 1.8 to 7.6), parenteral nutrition (OR =3.9; 95% CI, 1.6 to 9.6), tracheostomy (OR =4.4; 95% CI, 2.3 to 8.3) and colonization pressure >0.43 (OR =4; 95% CI, 1.2 to 5) as independently associated with the acquisition of *P. aeruginosa*, whereas exposure to fluoroquinolones for >3 days (OR =0.4; 95% CI, 0.2 to 0.8) was protective. In the whole series, prior exposure to carbapenems was independently associated with carbapenem resistance, and prior amikacin use predicted piperacillin-tazobactam, fluoroquinolone and multiple-drug resistance.

**Conclusions:**

In critical care settings with a high rate of antibiotic use, colonization pressure and non-antibiotic exposures may be the crucial factors for *P. aeruginosa* acquisition, whereas fluoroquinolones may actually decrease its likelihood. For the acquisition of strains resistant to piperacillin-tazobactam, fluoroquinolones and multiple drugs, exposure to amikacin may be more relevant than previously recognized.

**Electronic supplementary material:**

The online version of this article (doi:10.1186/s13054-015-0916-7) contains supplementary material, which is available to authorized users.

## Introduction

Previous exposure to antibiotics is considered an imperative risk factor for the acquisition of *Pseudomonas aeruginosa* and the subsequent development of infection [1]. According to the classical paradigm, non-antipseudomonal agents would promote acquisition of any *P. aeruginosa* strain [2,3], whereas drugs with antipseudomonal activity would select those resistant to the particular class of antimicrobial drug used [4]. Resistance acquisition driven by exposure to antipseudomonal agents can be reached by either selecting mutants in patients previously colonized or infected by susceptible phenotypes [5,6] or promoting selection of an already resistant strain [7]. Many study researchers have reported that prior exposure to a given antipseudomonal agent is associated with the acquisition of strains resistant to it [4,8-16], to unrelated agents [8-10,14,17-20] or to multiple drugs [13,21-27]. However, not enough data have been provided to ascertain which of the above-mentioned processes is preferentially involved [26,28].

There are discrepancies regarding the magnitude of the risk of resistance acquisition associated with the different antipseudomonal agents. In patients previously colonized or infected by *P. aeruginosa*, carbapenems and fluoroquinolones may have a greater tendency to select resistant mutants than other agents [5,6,29,30]. In addition, prior exposure to fluoroquinolones or carbapenems has commonly been associated with the acquisition of strains resistant to unrelated antibiotics and multiple drugs [10,15,17-22,24,26,31]. However, there are some exceptions. In a case–control study [24], cephalosporins and aminoglycosides (but not quinolones) were the main predictors of a multidrug-resistant (MDR) phenotype, and, in a cohort study [28], quinolones were protective against the acquisition of *P. aeruginosa* and had no role in the acquisition of resistant phenotypes.

Part of the discrepancies among studies regarding the role of previous use of antibiotics on *P. aeruginosa* resistance may be due to local differences in transmission rates, because exposure to antipseudomonal agents in previously non-colonized patients necessarily requires transmission from other patients or environmental sources to foster the acquisition of resistant strains. However, variables influencing transmission, such as colonization pressure [32], have rarely been taken into account in studies aimed at defining the influence of antibiotics on the acquisition of any *P. aeruginosa* or of strains with specific resistance phenotypes [2,3,14,33]. An accurate picture of the most meaningful epidemiological and exposure variables is essential to designing effective control measures directed at curbing the increasing incidence of the resistance of this important pathogen.

During a 3-year period, we were able to systematically obtain multisite surveillance cultures from patients admitted to a medical intensive care unit (ICU). This allowed us to investigate in detail the factors associated with the acquisition of *P. aeruginosa* and its different resistance phenotypes, taking into account both significant exposures (including antibiotics) and colonization pressure.

## Materials and methods

### Study population

From February 2006 to December 2008, all patients admitted to an 8-bed adult medical ICU of a 700-bed university hospital who stayed in the unit for at least 3 days (72 hours) were prospectively included in the study. The study unit has two individual rooms and a central space with six cubicles, and it is the reference unit for critically ill medical patients from the internal medicine, haematology, oncology and infectious diseases wards.

After a previous pilot experience [34], the director of the study unit decided to implement a mixing and cycling strategy of antibiotic use on a regular basis. To evaluate this policy, a prospective study of systematic screening for the detection of resistant or potentially resistant microorganisms was carried out during the first 3 years of its implementation. The present study was an analysis using clinical and microbiological data collected during the prospective screening program with the aim of investigating the risk factors for *P. aeruginosa* acquisition. The study protocol was approved by the Research Ethics Committee of the University Hospital Clinic of Barcelona, which waived the requirement of informed consent (approval reference number 2616).

### Microbiological procedures

Swabbing of nares, pharynx and rectum, as well as respiratory secretions (tracheobronchial aspirate, bronchoscopic samples or sputum), were obtained within 48 hours of admission and thrice weekly thereafter until discharge or the first 2 months of the ICU stay. Other clinical samples were obtained as deemed necessary by the attending physician. Samples were cultured in conventional agar media. No environmental cultures were taken. Susceptibility testing was done by using a microdilution technique according to Clinical and Laboratory Standards Institute guidelines [35]. For the purpose of analysis, intermediate susceptibility was considered as resistance. Molecular typing was performed by pulse-field gel electrophoresis as previously described [36]. Resistance to multiple antibiotics was defined as MDR, extensively drug-resistant (XDR) or pandrug-resistant (PDR) as described elsewhere [37].

### Clinical variables

Demographics, clinical variables, severity scores (Acute Physiology and Chronic Health Evaluation (APACHE) II and Sequential Organ Failure Assessment (SOFA)) upon admission and exposures during ICU stay were prospectively collected from all admitted patients as previously described. These data are shown in Table 1 [34].

**Table 1:** **Patient characteristics on admission, exposures during the ICU stay and outcomes of the entire population** ^**a**^

**Characteristics on admission**	**Total (N =850)**
Age (yr)	59.8 (17.3)
Male sex	519 (61.1)
Pre-ICU stay (days)	4.6 (12.2)
Prior antibiotic (≤1 mo)	258 (30.4)
APACHE II score	20 (6.5)
SOFA score	6.4 (3.6)
Shock on admission	151 (17.8)
Reason for admission	
Infection	486 (57.2)
CNS disease	99 (11.6)
Postsurgical	80 (9.4)
Cardiovascular disease	66 (7.8)
Respiratory disease	28 (3.3)
Others	91 (10.7)
Underlying diseases	
Diabetes mellitus	157 (18.5)
Haematological malignancy	114 (13.4)
Solid malignancy	85 (10)
COPD	138 (16.2)
Others^b^	193 (19.2)
Prior corticosteroids (≤1 mo)	156 (18.4)
Immunosuppressive therapy	90 (10.6)
Exposures during ICU stay	
Central venous catheter	833 (98)
Bladder catheter	800 (94.1)
Nasogastric tube	573 (67.4)
Enteral nutrition	253 (29.8)
Parenteral nutrition	177 (20.8)
Orotracheal intubation	511 (60.1)
Tracheostomy	158 (18.6)
Endoscopy	121 (34.2)
Surgery	189 (22.2)
Renal replacement therapies	77 (9.1)
Packed red blood cell transfusion	291 (34.2)
Any antibiotic	746 (87.8)
Any non-antipseudomonal antibiotic	593 (69.8)
Any antipseudomonal antibiotic:	576 (67.8%)
Carbapenem	281 (33.1)
Quinolone	306 (36)
Ceftazidime	103 (12.1)
Piperacillin-tazobactam	180 (21.2)
Amikacin	52 (6.1)
Outcomes	
Length of stay (days)	9.5 (10.3)
In-ICU mortality	117 (13.8)
In-hospital mortality	201 (23.6)

^a^APACHE II, Acute Physiology and Chronic Health Evaluation II; CNS, Central nervous system; COPD, Chronic obstructive pulmonary disease; ICU, Intensive care unit; SOFA, Sequential Organ Failure Assessment. ^b^Others include patients with HIV infection, hepatic cirrhosis, renal failure and heart failure. Categorical variables are expressed as number of patients (%) and continuous variables as mean (standard deviation).

### Antibiotic use

For the duration of the study, a policy of consecutive mixing and cycling periods of three classes of antipseudomonal agents (meropenem, ceftazidime/piperacillin-tazobactam and ciprofloxacin/levofloxacin) was implemented in the study unit. Each period lasted 4.5 months. During mixing, a different antipseudomonal antibiotic class was prescribed to each consecutive patient. Cycling periods were divided in three consecutive 6-week intervals in which a different antibiotic class was given to every patient. The decision to provide antipseudomonal antibiotics was made by the attending physician based on clinical judgment. Amikacin in a once-daily dose was the aminoglycoside favoured for antipseudomonal antibiotic coverage, but its administration as monotherapy or for >5 days was discouraged. The decision to administer combination treatment with a β-lactam and a fluoroquinolone or amikacin was also made by the attending physician, and, in accordance with current protocols, it was encouraged only for patients with severe sepsis or septic shock.

### Epidemiological variables

The results of surveillance cultures were communicated to the attending physician either when they yielded a microorganism requiring contact precautions according to current isolation practices in the hospital (methicillin-resistant *Staphylococcus aureus* (MRSA); vancomycin-resistant enterococci (VRE); enteric Gram-negative bacilli producing extended-spectrum β-lactamases; *P. aeruginosa* resistant to at least three classes of antipseudomonal agents, considering ceftazidime and piperacillin-tazobactam or ciprofloxacin and levofloxacin as single classes) or when an outbreak was suspected. Contact precautions implied the transfer to an individual room when available and, in any case, the wearing of gowns and gloves when entering the cubicle or room. Patients with prior MRSA, MDR Gram-negative bacilli and VRE were automatically identified by an electronic tag on admission, but preventive isolation based on risk factors was never performed. Hand hygiene was primarily based on alcohol-based hand rubs. Decolonization with mupirocin was carried out only in patients with MRSA present exclusively in nares. Chlorhexidine was used for oral hygiene, but not for body bathing. Selective decontamination of the digestive tract or any additional practice, such as the use of extraordinary prophylactic antibiotics (except as clinically recommended in neutropenic, cirrhotic or HIV patients), was not performed during the study. There were no changes in isolation or hand hygiene practices during the study period.

### Definitions

*Colonization* was defined as the isolation of *P. aeruginosa* from a surveillance culture or non-sterile clinical sample. Patients with *P. aeruginosa* isolated within 48 hours of ICU admission were considered to be colonized upon admission. Organisms isolated 48 hours after admission in patients with previous negative specimens were considered as ICU-acquired. Infection was considered the reason for admission when the organic failure leading to critical care was understood to be a direct consequence of either the dysfunction of the infected organ or sepsis. *Acquisition of resistance* was defined as the isolation of a resistant organism in a patient with a previous sensitive strain or prior negative cultures. *Emergence of resistance* to a given antibiotic refers to the conversion of a genotypically defined strain from susceptible to non-susceptible; hence, these isolates were also included in the previous definition. Cross-transmission was considered to have occurred when a patient acquired a pulsotype identical to that of an isolate previously found in a patient who stayed in the unit during the same period. Colonization pressure was estimated as the average of the daily proportion of colonized patients (number of patients colonized divided by number of patients in the unit on a given day) from the day of admission until the day before acquisition of the microorganism or until discharge if the patient did not acquire the microorganism [38]. Time at risk was the number of days until the detection of the microorganism and/or resistance in patients who acquired it and the whole length of ICU stay if the patient did not acquire it. *Exposure to antibiotics* meant at least 24 hours of treatment.

### Statistical analysis

For continuous variables, means (with standard deviations) or medians (with interquartile ranges (IQRs)) were used as measures of central tendency (dispersion). Denominators in proportions were always ‘number of patients’. Proportions were compared by using the χ^2^ test or Fisher’s exact test, and continuous variables were compared by using the *t*-test or Mann–Whitney *U* test. Multivariable logistic regression analysis (step-forward procedure) was used to evaluate characteristics associated with the acquisition of *P. aeruginosa* and acquisition of resistance to ceftazidime, piperacillin-tazobactam, carbapenems and quinolones. For the purpose of analysing the risk factors associated with the acquisition of *P. aeruginosa*, patients colonized or infected with this microorganism upon admission were excluded, owing to uncertainty about the ability to detect their new episodes of acquisition. However, the whole cohort was considered when we analysed the risk factors for the acquisition of resistance to the different antipseudomonal antibiotics, because resistance could emerge in strains of *P. aeruginosa* present upon admission. Age, APACHE II score and SOFA score were introduced into the models as dichotomous variables, taking the median as the cutoff value, whereas colonization pressure was dichotomized by the highest observed value (95th percentile). In multivariate models predicting the acquisition of strains resistant to each antipseudomonal agent and multiple drugs, a cutoff of 72 hours was used to dichotomize antibiotic exposure because in previous studies it appeared to be the best time span for defining the minimal duration of exposure associated with resistance [39,40]. Variables with a *P*-value <0.3 in the univariate analysis were introduced into the multivariate model. Calculations were done using the IBM SPSS version 20.0 statistical software package (IBM, Armonk, NY, USA).

## Results

During the 35-month study period, a total of 850 patients were hospitalized in the unit for 72 hours or more. Patient characteristics and exposures are shown in Table 1, and a detailed description of the reasons for admission is provided in Additional file 1.

In regard to antibiotic exposure, 751 patients (88.3%) received an antibiotic, 562 (66.1%) of which were antipseudomonal agents. The median daily dosages of antipseudomonal antibiotics were 6 g for ceftazidime, 3 g for carbapenems (meropenem or imipenem; there was no exposure to ertapenem), 12 g for piperacillin-tazobactam, 1,200 mg for ciprofloxacin, 500 mg for levofloxacin and 1 g for amikacin. The median days (IQR) of exposure to antipseudomonal antibiotics were 6 (3 to 11) for ceftazidime, 6 (3 to 10) for carbapenems, 5 (3 to 8) for piperacillin-tazobactam, 6 (3 to 10) for ciprofloxacin, 4 (3 to 8) for levofloxacin and 4 (2 to 10) for amikacin. The median (IQR) length of ICU stay was 5 (4 to 10) days.

During the study period, a total of 9,561 surveillance samples were obtained and cultured, of which 1,646 proceeded from the lower respiratory tract, 2,664 from the pharynx, 2,690 from the nares and 2,561 from the rectum. The mean numbers per included patient was 3.2 for nasal swabs, 3.1 for pharyngeal swabs, 3 for rectal swabs and 1.9 for respiratory samples (3.3 in intubated patients).

A total of 68 patients (8%) were colonized with *P. aeruginosa* upon admission, and of the remaining 782, 104 (13.3%) acquired at least one strain of *P. aeruginosa* during their ICU stay (4 patients acquired 2 different strains). Acquired isolates belonged to 79 distinct pulsotypes, of which 7 were obtained from more than 1 patient (from 2 to 20). The more numerous cluster, which included 20 patients, corresponded to a strain that had a XDR phenotype on 16 occasions. Of the 104 patients who acquired *P. aeruginosa*, in 20 (19.2%) acquisition was due to cross-transmission (13 of the XDR genotype) and in the remaining patients the origin was unknown. The sites of primary detection were the rectum in 57 cases (54.8%), the nares or pharynx in 16 (15.3%), the lower respiratory tract in 10 (9.6%), more than one of these sites in 19 (18.2%) and other sites in 2 (1.9%). Of the 57 patients with initial unique rectal colonization, 18 (31.5%) had subsequent nasopharyngeal (n =3) or lower respiratory tract colonization (n =15). In all, 48 patients (46.1%) eventually had *P. aeruginosa* isolated from a lower respiratory sample (in 24 as a first site of colonization and in 24 as a secondary one), of whom 13 (27%) had pneumonia (11 ventilator-associated). Detection in tracheal aspirates or sputum preceded pneumonia in seven patients for a median of 3 days (range, 1 to 14), and it was coincidental with its clinical diagnosis in the remaining six patients. Pneumonia occurred more frequently in patients with first detection of *P. aeruginosa* in the lower respiratory (8 of 24 (33.3%)) than in other sites (5 of 80 (6.2%)) (*P* =0.002). Other infections diagnosed in the 104 patients who acquired *P. aeruginosa* were ventilator-associated tracheobronchitis in 9, catheter-related bacteraemia in 3, primary bacteraemia in 2 and a surgical wound infection in 1. Tracheobronchitis was equally common in patients with first detection of *P. aeruginosa* in the lower respiratory tract (2 of 24 (8.3%)) than in other sites (7 of 80 (8.7%) (*P* =1), whereas the 6 other infections occurred in patients in whom *P. aeruginosa* was first detected outside the lower respiratory tract. Sites of primary and secondary acquisition are shown in detail in Additional file 2.

### Risk factors for acquisition of *Pseudomonas aeruginosa*

The acquired strains were susceptible to all antipseudomonal antibiotics in 56 patients (53%), PDR in 1 (1%), XDR in 17 (16%), MDR in 4 (4%) and resistant to 1 or 2 groups of antipseudomonal antibiotics in 27 (26%). Upon acquisition, resistance to carbapenems, piperacillin-tazobactam, ceftazidime, fluoroquinolones and amikacin was observed in 39 (37%), 19 (18%), 30 (29%), 29 (28%) and 1 (1%) strains, respectively.

The univariate analysis of the relationship between patient’s characteristics or exposures and the acquisition of *P. aeruginosa* is shown in Table 2. Multivariate analysis selected shock, orotracheal intubation, enteral nutrition for ≤3 days, parenteral nutrition for ≤3 days, tracheostomy and colonization pressure >0.43 as being independently associated with the acquisition of *P. aeruginosa*, whereas exposure to fluoroquinolones for >3 days was protective. The complete model is shown in Table 3.

**Table 2:** **Relationship between** ***Pseudomonas aeruginosa*** **acquisition, characteristics on admission and exposures in the ICU** ^**a**^

**Characteristics**	**Acquisition of** ***P. aeruginosa***	**No acquisition of** ***P. aeruginosa***	**OR**	***P*** **-value**
	**(n =104)**	**(n =678)**	**(95% CI)**	
Male sex	36 (34.6)	270 (39.8)	0.8 (0.5 to 1.2)	0.3
Pre-ICU hospital stay >3 days	36 (34.6)	143 (21.1)	1.98 (1.3 to 3.1)	0.002
Underlying diseases				
Neutropenia	4 (3.8)	15 (2.2)	1.77 (0.6 to 5.4)	0.3
Haematological malignancy	10 (9.6)	93 (13.7)	0.67 (0.3 to 1.3)	0.2
Liver cirrhosis	8 (7.7)	22 (3.2)	2.48 (1.1 to 5.7)	0.03
Other conditions on admission				
Prior antibiotic (≤1 mo)	38 (36.5)	194 (28.6)	1.44 (0.9 to 2.2)	0.1
Shock	29 (27.9)	105 (15.5)	2.11 (1.3 to 3.4)	0.002
Reason for admission				
Infection	67 (64.4)	369 (54.4)	1.52 (1 to 2.3)	0.1
Postsurgical	1 (1)	78 (11.5)	0.07 (0 to 0.5)	0.001
Severity scores				
APACHE II score ≥20	63 (60.6)	328 (48.4)	1.64 (1.1 to 2.5)	0.02
SOFA score ≥7	64 (61.5)	306 (45.1)	1.95 (1.3 to 3)	0.002
Non-antibiotic exposures				
Days at risk, median (IQR)	6 (4 to 11)	5 (3 to 8)	–	0.01
Colonization pressure^b^ >0.43	5 (4.8)	12 (1.8)	2.8 (1 to 8.1)	0.05
Mixing periods	49 (47.1)	281 (41.5)	0.8 (0.5 to 1.2)	0.2
CVC >3 days	80 (76.9)	472 (69.6)	1.45 (0.9 to 2.4)	0.1
Bladder catheterization				
No	2 (1.9)	45 (6.6)	1	
1 to 3 days	23 (22.1)	167 (24.6)	3.1 (0.7 to 13.6)	0.1
>3 days	79 (76)	466 (68.7)	3.8 (0.9 to 16)	0.1
Intubation				
No	15 (14.4)	306 (45.1)	1	
1 to 3 days	23 (22.1)	187 (27.6)	2.5 (1.3 to 4.9)	0.01
>3 days	66 (63.5)	185 (27.3)	7.3 (4 to 13.1)	<0.001
Enteral nutrition				
No	47 (45.2)	18 (76.4)	1	
1 to 3 days	21 (20.2)	40 (5.9)	5.8 (3.2 to 10.6)	<0.001
>3 days	36 (34.6)	120 (17.7)	3.3 (2.1 to 5.3)	<0.001
Parenteral nutrition				
No	65 (62.5)	566 (83.5)	1	
1 to 3 days	10 (9.6)	21 (3.1)	4.1 (1.9 to 9.2)	<0.001
>3 days	29 (27.9)	91 (13.4)	2.8 (1.7 to 4.5)	<0.001
Tracheostomy	48 (46.2)	84 (12.4)	6.06 (3.9 to 9.5)	<0.001
Endoscopy	21 (20.2)	77 (11.4)	1.97 (1.2 to 3.4)	0.01
Surgery	18 (17.3)	68 (10)	1.88 (1.1 to 3.3)	0.03
Blood transfusion	45 (43.3)	198 (29.2)	1.85 (1.2 to 2.8)	0.004
Antibiotic exposures during the ICU stay				
Fluoroquinolone				
No	74 (71.2)	447 (65.9)	1	
1 to 3 days	9 (8.7)	85 (12.5)	0.6 (0.3 to 1.3)	0.2
>3 days	21 (20.2)	146 (21.5)	0.9 (0.5 to 1.5)	0.6
Carbapenem				
No	64 (61.5)	483 (71.2)	1	
1 to 3 days	10 (9.6)	64 (9.4)	1.2 (0.6 to 2.4)	0.7
>3 days	30 (28.8)	131 (19.3)	1.7 (1.1 to 2.8)	0.02
Ceftazidime				
No	94 (90.4)	618 (91.2)	1	
1 to 3 days	0 (0)	23 (3.4)	–	1
>3 days	10 (9.6)	37 (5.5)	1.8 (0.9 to 3.7)	0.1
Piperacillin-tazobactam				
No	79 (76)	549 (81)	1	
1 to 3 days	8 (7.7)	45 (6.6)	1.2 (0.6 to 2.7)	0.6
>3 days	17 (16.3)	84 (12.4)	1.4 (0.8 to 2.5)	0.2
Amikacin				
No	99 (95.2)	655 (96.6)	1	
1 to 3 days	1 (1)	13 (1.9)	0.5 (0.1 to 3.9)	0.5
>3 days	4 (3.8)	10 (1.5)	2.6 (0.8 to 8.6)	0.1
Any antibiotic	102 (98.1)	578 (85.3)	8.82 (2.1 to 36.3)	<0.001
Any antipseudomonal antibiotic				
No	30 (28.8)	246 (36.3)	1	
1 to 3 days	22 (21.2)	188 (27.7)	1 (0.5 to 1.7)	0.9
>3 days	52 (50)	244 (36)	1.7 (1.1 to 2.8)	0.02
Any non-antipseudomonal antibiotic				
No	20 (19.2)	231 (34.1)	1	
1 to 3 days	20 (19.2)	146 (21.5)	1.6 (0.8 to 3)	0.2
>3 days	64 (61.5)	301 (44.4)	2.5 (1.4 to 4.2)	<0.001

^a^APACHE II, Acute Physiology and Chronic Health Evaluation II; CI, Confidence interval; CVC, Central venous catheter; ICU, Intensive care unit; IQR, Interquartile range; OR, Odds ratio; SOFA, Sequential Organ Failure Assessment. Variables are expressed in terms of frequency as number of patients (%) and in terms of duration as median days (IQR). ^b^Value corresponding to the 95th percentile. Variables with *P* ≤0.3 introduced in the multivariate analysis and not shown include the following: infections on admission (pneumonia, urinary tract infection and primary bacteraemia), arterial catheter, nasogastric tube, corticosteroids, glycopeptides, clindamycin, macrolide, trimethoprim-sulphamethoxazole, linezolid, fluconazole, other penicillins and other cephalosporins. Variables with a *P*-value >0.3 are not shown and include the following: age; bone marrow transplant; solid organ transplant; solid organ cancer; haemodialysis; HIV infection; heart failure; chronic obstructive pulmonary disease; diabetes; prior corticosteroid and immunosuppressive therapy; admission within the previous year; respiratory, cardiovascular, central nervous system and other diseases as reasons for admission; catheter-related bacteraemia as prevalent infection; and renal replacement therapy.

**Table 3:** **Multivariate analysis of factors associated with** ***Pseudomonas aeruginosa*** **acquisition during ICU stay** ^**a**^

**Variables**	**OR (95% CI)**	***P*** **-value**
Shock	2.1 (1.2 to 3.7)	0.01
Colonization pressure >0.43	4 (1.2 to 12.8)	0.02
Intubation		
No	Reference group	
1 to 3 days	2.5 (1.2 to 5)	0.01
>3 days	3.6 (1.7 to 7.5)	0.001
Enteral nutrition		
No	Reference group	
1 to 3 days	3.6 (1.8 to 7.6)	0.001
>3 days	1 (0.5 to 2.1)	1
Tracheostomy	4.4 (2.3 to 8.3)	<0.001
Parenteral nutrition		
No	Reference group	
1 to 3 days	3.9 (1.6 to 9.6)	0.003
>3 days	1.1 (0.6 to 2.2)	0.7
Prior exposure to fluoroquinolones		
No	Reference group	
1 to 3 days	0.5 (0.2 to 1.2)	0.2
>3 days	0.4 (0.2 to 0.8)	0.01

^a^CI, Confidence interval; ICU, Intensive care unit; OR, Odds ratio. Hosmer-Lemeshow goodness-of-fit test value of 9.7 (*P* =0.2).

### Risk factors for the acquisition of resistance to antipseudomonal agents

The number of patients in whom *P. aeruginosa* resistant to the different antipseudomonal antibiotics was isolated during the ICU stay, the resistance status when first isolated and the number of acquisitions due to cross-transmissions are stated in Table 4. In most cases, the resistance phenotype was acquired as such and did not emerge from a susceptible one. Only one strain acquired as susceptible was the result of cross-transmission, whereas this was observed in 41% to 58% of the strains acquired as resistant to the different antipseudomonal β-lactams or fluoroquinolones.

**Table 4:** ***Pseudomonas aeruginosa*** **resistance to antibiotics when first detected and cross-transmission cases** ^**a**^

**Resistant antibiotics**	**Upon admission as susceptible**	**Acquired in ICU as susceptible**	**Acquired in ICU as resistant**
Ceftazidime (n =40)	2 (5)	8 (20) [0]	30 (75) [15]
Carbapenems (n =46)	2 (4)	5 (11) [0]	39 (85) [16]
Piperacillin-tazobactam (n =31)	1 (3)	11 (35) [1]	19 (61) [11]
Quinolones (n =39)	2 (5)	8 (21) [0]	29 (74) [15]
Amikacin (n =1)	0	0	1 (100) [1]
MDR (n =31)	2 (6)	7 (23) [0]	22 (71) [14]

^a^ICU, Intensive care unit; MDR, Multidrug-resistant. Number of patients in whom *P. aeruginosa* resistant to the different antipseudomonal antibiotics was isolated during ICU stay, resistance status of the strains when first detected (percentage) and number of cases due to cross-transmission [in brackets].

Univariate analysis of the association of prior exposure to carbapenems, ceftazidime, piperacillin-tazobactam, fluoroquinolones and amikacin with acquisition of resistance to the different antipseudomonal antibiotics and multiple drugs is shown in Additional file 3.

In multivariate analysis, carbapenem exposure for more than 3 days was associated with acquisition of resistance to itself, and amikacin exposure for more than 3 days was associated with acquisition of resistance to piperacillin-tazobactam and fluoroquinolones as well as MDR. Exposure to fluoroquinolones, piperacillin-tazobactam or ceftazidime was not associated with acquisition of resistance to themselves or to other antipseudomonal agents. Complete models are shown in Additional file 4.

Emergence of resistance to a given antipseudomonal agent from a previous susceptible strain after exposure to itself occurred in 4 (20%) of 20 patients exposed to ceftazidime (vs 8 (8%) of 106 non-exposed; *P* =0.1), 6 (46%) of 13 exposed to carbapenems (vs 1 (3%) of 95 non-exposed; *P* <0.001), 3 (15%) of 20 exposed to piperacillin-tazobactam (vs 9 (8%) of 117 non-exposed; *P* =0.3) and 8 (29%) of 28 exposed to fluoroquinolones (vs 2 (3%) of 94 non-exposed; *P* <0.001).

## Discussion

The main findings of this study are the following: (1) colonization pressure and several patient conditions or instrumentations seem to be more relevant risk factors than exposure to antibiotics for the acquisition of *P. aeruginosa*; (2) exposure to fluoroquinolones (levofloxacin or ciprofloxacin) for >3 days was protective against the acquisition of this pathogen; (3) exposure to carbapenems predicted resistance to themselves; and (4) amikacin exposure was associated with the acquisition of resistance to piperacillin-tazobactam, quinolones and multiple drugs.

Whenever cross-transmission is involved in the acquisition of a given microorganism, it is expected that colonization pressure should be a relevant risk factor. Although defined in different ways, colonization pressure has been independently associated, in the ICU setting, with the acquisition of MRSA [41], VRE [38], *Clostridium difficile* [42], *Acinetobacter baumannii* [33,43] and *P. aeruginosa* [16,33,44]. However, in none of the MRSA studies, and in only some on *A. baumannii* [43,45] or *P. aeruginosa* [16], was adjustment for prior antibiotic exposure performed. Some reports indicate that there is an interaction between colonization pressure and antibiotics. In one study in which investigators searched for predictors of *P. aeruginosa* acquisition in the ICU [44], prior exposure to ≥3 days of non-antipseudomonal antibiotics was a significant risk factor only when there was at least one colonized patient in the unit. This fact supports the notion that, in previously non-colonized patients, antibiotics cannot promote acquisition of resistance without relying on transmission. In another study on imipenem-resistant *A. baumannii* acquisition, antimicrobials were found to be a risk factor only for patients admitted during periods in which colonization pressure was low [45], suggesting that the role of antibiotics may be relatively more important when there are fewer opportunities for patient-to-patient transmission. Our data indicate that colonization pressure, measured as originally described [38], was an independent risk factor for the acquisition of *P. aeruginosa* in a critical care setting where most patients were exposed to antibiotics (87% to any drug and 64.7% to an antipseudomonal antibiotic) and 19% of the acquisition episodes were due to cross-transmission. We think that having a daily colonization pressure chart for the main pathogens of interest in a given ICU may therefore be useful for quantifying the risk of new acquisitions and establish the appropriate control measures aimed at preventing this untoward event.

In regard to the role of antibiotics, the most striking finding of the present study was that fluoroquinolones were actually protective for the acquisition of *P. aeruginosa* and rather neutral for the acquisition of its resistance phenotypes. In critically ill patients, in the few previous studies in which researchers have specifically investigated *P. aeruginosa* acquisition, findings have been that fluoroquinolones are protective against it in the pharynx [46] or in any site [28]. In hospital-wide studies, levofloxacin (but not ciprofloxacin) has been reported to be protective against nosocomial infection due to fluoroquinolone-susceptible *P. aeruginosa* [12] and also against Gram-negative bacilli (including *P. aeruginosa*) colonization or infection with chromosomally mediated cephalosporin resistance [47]. All these data, including ours, suggest that fluoroquinolones, when administered to critically ill patients not previously colonized by *P. aeruginosa*, may decrease the burden of new acquisition, even in settings where the prevalence of resistance to fluoroquinolones is around 29%. Even more surprising is the persistent inability of our group to find an independent association between prior exposure to antipseudomonal quinolones and the acquisition of a particular resistant phenotype or MDR [28]. A plethora of previous case–control or cohort studies have linked this antibiotic class with resistance to themselves [11-13], to antipseudomonal β-lactams [10,17-19] or to multiple drugs [21,23,25-27]. We do not have a satisfactory explanation of this discrepancy, but the fact that we found such association (of prior use of quinolones with resistance to themselves and MDR) in the univariate analysis, but not in the multivariate analysis, enhances the absolute need of careful consideration of potential confounders. In contrast to fluoroquinolones, the present data confirm the involvement of prior carbapenem use in acquisition of resistance to itself [9,14-16]. It is of note that, in our experience, both quinolones and carbapenems had a higher propensity than ceftazidime or piperacillin-tazobactam to select resistance to themselves when administered to patients previously colonized with susceptible strains. These data suggest that, in critically ill patients not colonized by *P. aeruginosa* or at low risk of carriage, quinolones may be safer than carbapenems in terms of risk of acquisition of resistant strains and may even lower the burden of *P. aeruginosa*. In other circumstances, ceftazidime and piperacillin-tazobactam can be associated with a lower risk of resistance acquisition because they apparently have a lesser tendency than carbapenems to select resistance in patients previously colonized. However, when trying to select an appropriate empirical antibiotic regimen in patients with severe sepsis, it remains appropriate to avoid the administration of a recently used antipseudomonal antibiotic, to take into consideration the local rates of *P. aeruginosa* resistance if this pathogen is an issue and to consider the risk of other MDR microorganisms [48].

In the present study, previous administration of amikacin was associated with the acquisition of resistance to fluoroquinolone, piperacillin-tazobactam and multiple drugs in the multivariate analysis. A small number of prior studies have also noted an association between amikacin or aminoglycosides and acquisition of *P. aeruginosa* resistant to imipenem, [9,14] ceftazidime [9], piperacillin-tazobactam [8] and multiple drugs [13,24]. We consider that such an association cannot be attributed to the selection of MexXY overproducers [49], because no increase in amikacin minimum inhibitory concentrations were observed. Although relying on multivariate analysis, there is still room for non-casual associations; hence, these should be validated in other studies.

Many of the non-antibiotic-related confounders in our study denoting exposure to medical devices, severity of the underlying condition and duration of critical status have previously been reported as risk factors for the acquisition of any *P. aeruginosa* or strains with single-drug resistance or MDR phenotypes [3,28,50].

This study has several limitations, the most obvious being that it was conducted in a single medical ICU whose results may not be applicable to other settings with different epidemiological characteristics. In addition, the number of outcomes regarding the different resistance phenotypes and MDR were scarce, thus increasing uncertainty about the quality of the multivariate models. On the other hand, as sensitivity of surveillance cultures was probably not complete, the colonization status of some patients could have been misclassified. Finally, information about compliance with infection control measures during the study period and surveillance cultures from the environment was not available.

## Conclusions

In ICU settings with a high rate of antibiotic use, colonization pressure and non-antibiotic exposures may be more important than antibiotics as determinants of *P. aeruginosa* acquisition; antipseudomonal quinolones may actually prevent it; and, regarding the acquisition of resistance to selected β-lactams (piperacillin-tazobactam), fluoroquinolones and multiple drugs, exposure to amikacin may be a more crucial risk factor than previously recognized.

## Key messages

- In ICU settings with a high rate of antibiotic use, colonization pressure and non-antibiotic exposures are the main determinants of *P. aeruginosa* acquisition.
- Fluoroquinolones may prevent the acquisition of *P. aeruginosa.*
- Carbapenem exposure is associated with acquisition of carbapenem resistance, and amikacin exposure is associated with acquisition of resistance to piperacillin-tazobactam and fluoroquinolones and MDR.
- In previously sensitive strains of *P. aeruginosa*, emergence of resistance occurs more frequently after exposure to carbapenems and fluoroquinolones than to ceftazidime or piperacillin-tazobactam.

## Additional files

Additional file 1:
**Reasons for admission of the entire cohort (850 patients).**
Additional file 2:
**Sites of primary and secondary** ***P. aeruginosa*** **acquisition.**
Additional file 3:
**Relationship between acquisition of resistance to antipseudomonal antibiotics and prior exposure to different agents.**
Additional file 4:
**Multivariate analysis of factors associated with acquisition of resistance to each antipseudomonal agent and MDR.**

## Abbreviations

APACHE: Acute Physiology and Chronic Health Evaluation
CI: Confidence interval
CNS: Central nervous system
COPD: Chronic obstructive pulmonary disease
CVC: Central venous catheter
ICU: Intensive care unit
IQR: Interquartile range
MDR: Multidrug-resistant
MRSA: Methicillin-resistant *Staphylococcus aureus*
OR: Odds ratio
PDR: Pandrug-resistant
SOFA: Sequential Organ Failure Assessment
VRE: Vancomycin-resistant enterococci
XDR: Extensively drug-resistant
