# Supplementary material for: Acquisition of Pseudomonas aeruginosa and its resistance phenotypes in critically ill medical patients: role of colonization pressure and antibiotic exposure
Source: Crit Care. 2015 May 4;19(1):218. doi: 10.1186/s13054-015-0916-7 (PMC4432505; doi:10.1186/s13054-015-0916-7)
Supplement: Additional file 1: — Reasons for admission of the entire cohort (850 patients). [file 13054_2015_916_MOESM1_ESM.docx]

Additional file 1. Reasons for admission of the entire cohort (850 patients).

| **Reasons for admission** | **Number of patients (%)** | **Need for intubation (%)** |
| --- | --- | --- |
| **Infection** | 486 | 273 (56.1) |
| Community-acquired pneumonia | 160 (32.9) | 92 (57.5) |
| Hospital-acquired pneumonia | 74 (15.2) | 57 (77) |
| Intraabdominal | 61 (12.5) | 37 (52.1) |
| Acute exacerbation of COPD | 55 (11.3) | 28 (50.9) |
| Central nervous system | 26 (5.3) | 15 (57.6) |
| Urinary tract | 23 (4.7) | 2 (8.6) |
| Skin and soft tissues | 16 (3.3) | 11 (68.7) |
| Endocarditis | 11 (2.2) | 9 (81.8) |
| Invasive aspergillosis or candidiasis | 8 (1,6) | 5 (62.5) |
| Primary bacteremia | 7 (1.4) | 0 |
| Catheter-related bacteremia | 4 (0.8) | 3 (75) |
| Miliary tuberculosis | 3 (0,6) | 2 (66.6) |
| Severe malaria | 3 (0,6) | 1 (33.3) |
| Other sources | 20 (4.1) | 9 (45) |
| Sepsis of unknown origin | 15 (3) | 2 (13.3) |
| **Central nervous system disease** | 99 | 68 (68.6) |
| Status epilepticus | 25 (25.3) | 17 (68) |
| Metabolic or toxic coma | 20 (20.2) | 15 (75) |
| Intraparenchymal hemorrhage | 19 (19.2) | 16 (84.2) |
| Subarachnoid hemorrhage | 15 (15.1) | 8 (53.3) |
| Ischemic stroke | 10 (8) | 8 (80) |
| Subdural hematoma | 4 (4) | 1 (25) |
| Guillain-Barré syndrome | 2 (2) | 1 (50) |
| Other | 4 (4) | 2 (50) |
| **Postsurgical** | 80 | 71 (88.7) |
| Coronary artery bypass | 36 (45) | 34 (94.4) |
| Valve replacement | 36 (45) | 32 (88.8) |
| Aortic aneurysm rupture | 2 (2.5) | 2 (100) |
| Gastrointestinal resection | 3 (3.8) | 1 (33.3) |
| Other | 3 (3.8) | 2 (66.6) |
| **Cardiovascular disease** | 66 | 43 (65.1) |
| Congestive heart failure | 16 (24.2) | 8 (50) |
| Cardiac arrest | 15 (22.7) | 14 (93.3) |
| Acute myocardial infarction | 13 (19.6) | 7 (53.8) |
| Cardiogenic shock | 8 (12.1) | 5 (62.5) |
| Superior vena cava syndrome | 5 (7.6) | 4 (80) |
| Cardiac tamponade | 3 (4.5) | 1 (33.3) |
| Aortic aneurysm threat rupture | 2 (3) | 2 (100) |
| Life-threatening arrhythmia | 2 (3) | 1 (50) |
| Hypertensive emergency | 2 (3) | 1 (50) |
| **Respiratory disease** | 28 | 19 (67.8) |
| Bronchoaspiration | 4 (14.2) | 4 (100) |
| Alveolar hemorrhage | 3 (10.7) | 1 (33.3) |
| Pulmonary embolism | 3 (10.7) | 2 (66.6) |
| Miscellaneous acute respiratory  failure | 18 (64.2) | 12 (66.6) |
| **Other** | 91 | 37 (40.6) |
| Miscellaneous hepatic, pancreatic and  gastrointestinal disorders | 13 (14.2) | 6 (46.1) |
| Miscellaneous metabolic disorders | 20 (21.9) | 12 (60) |
| Miscellaneous acute intoxications | 29 (31.8) | 12 (41.3) |
| Other | 29 (31.8) | 7 (24.1) |
