# Supplementary material for: Acquisition of Pseudomonas aeruginosa and its resistance phenotypes in critically ill medical patients: role of colonization pressure and antibiotic exposure
Source: Crit Care. 2015 May 4;19(1):218. doi: 10.1186/s13054-015-0916-7 (PMC4432505; doi:10.1186/s13054-015-0916-7)
Supplement: Additional file 2: — Sites of primary and secondary P. aeruginosa acquisition. [file 13054_2015_916_MOESM2_ESM.docx]

Additional file 2. Sites of primary and secondary *P. aeruginosa* acquisition.

| **Site** | **Primary site of acquisition (multiple sites)** | **Secondary site of acquisition** | **Total no. of patients with site involvement at any time (multiple sites)** |
| --- | --- | --- | --- |
| Nares or pharynx | 34 (23)^a^ | 12 | 46 (39)^b^ |
| Lower respiratory | 24 (14)^c^ | 24 | 48 (43)^d^ |
| Rectum | 67 (10)^e^ | 8 | 75 (35)^f^ |
| Surgical wound | 1 | 0 | 1 (0) |
| Bloodstream | 1 | 0 | 1 (1)^g^ |

^a^ 11 patients had initial single site colonization, 1 in nares and 10 in pharynx. Multiple sites included: nares plus pharynx in 5 patients, pharynx plus lower respiratory in 3, pharynx plus rectal in 3, nares plus pharynx plus lower respiratory in 6, nares plus pharynx plus rectal in 2 and all four sites in 4. ^b^ Multiple sites included: nares plus pharynx in 3 patients, nares plus lower respiratory in 1, pharynx plus lower respiratory in 6, pharynx plus rectal in 2, nares plus pharynx plus lower respiratory in 5, nares plus pharynx plus rectal in 3, nares plus lower respiratory plus rectal in 1, pharynx plus lower respiratory plus rectal in 4 and all sites in 14. ^c^ Multiple sites included: lower respiratory plus pharynx in 3, lower respiratory plus rectal in 1, lower respiratory plus nares plus pharynx in 6 and all sites in 4. ^d^ Multiple sites included: lower respiratory plus pharynx in 6, lower respiratory plus rectal in 11, lower respiratory plus nares in 1, lower respiratory plus nares plus pharynx in 5, lower respiratory tract plus rectal plus pharynx or nares in 5, and all sites in 15. ^e^ Multiple sites included: rectal plus pharynx in 3, rectal plus lower respiratory in 1, rectal plus nares plus pharynx in 2, and all sites in 4. ^f^ Multiple sites included: rectal plus pharynx in 1, rectal plus lower respiratory in 11, rectal plus bloodstream in 1, rectal plus nares plus pharynx in 3, rectal plus nares plus lower respiratory in 1, rectal plus pharynx plus lower respiratory in 4, and all sites in 14. ^g^ Multiple sites include blood stream plus rectal.
