# Supplementary material for: Acquisition of Pseudomonas aeruginosa and its resistance phenotypes in critically ill medical patients: role of colonization pressure and antibiotic exposure
Source: Crit Care. 2015 May 4;19(1):218. doi: 10.1186/s13054-015-0916-7 (PMC4432505; doi:10.1186/s13054-015-0916-7)
Supplement: Additional file 3: — Relationship between acquisition of resistance to antipseudomonal antibiotics and prior exposure to different agents. [file 13054_2015_916_MOESM3_ESM.docx]

Additional file 3. Relation between acquisition of resistance to antipseudomonal antibiotics and prior exposure to different agents.

| **Acquisiton of resistance to** | **Patients number**  **(%)** | **Number (%) of patients previously exposed to** | | | | | | | | | | | | | | |
| --- | --- | --- | --- | --- | --- | --- | --- | --- | --- | --- | --- | --- | --- | --- | --- | --- |
|  |  | Ceftazidime | OR (IC) | p | Carbapenem | OR (IC) | p | PIP-TAZ | OR (IC) | p | Quino-  lones | OR (IC) | p | Ami-  kacin | OR (IC) | p |
| Ceftazidime  Yes  No | 40 (5)  810 (95) | 6 (15)  60 (7) | 2.2 (0.9-5.5) | 0.08 | 16 (40)  173 (21) | 2.5 (1.3-4.7) | 0.006 | 9 (23)  116 (14) | 1.7 (0.8-3.7) | 0.15 | 14 (35)  187 (23) | 1.8 (0.9-3.5) | 0.08 | 4 (10)  21 (3) | 2.0 (0.8-10.1) | 0.007 |
| Carbapenems  Yes  No | 46 (5)  804 (95) | 2 (4)  63 (8) | 0.5 (0.1-2.3) | 0.39 | 22 (48)  167 (21) | 3.5 (1.9-6.4) | <0.001 | 8 (17)  114 (14) | 1.3 (0.6-2.8) | 0.55 | 13 (28)  186 (23) | 1.3 (0.7-2.5) | 0.4 | 3 (7)  22 (3) | 2.5 (0.7-8.6) | 0.14 |
| PIP-TAZ  Yes  No | 31 (4)  819 (96) | 7 (23)  62 (8) | 3.6 (1.5-8.6) | 0.003 | 14 (45)  177 (22) | 3 (1.4-6.2) | 0.002 | 8 (26)  118 (14) | 2.1 (0.9-4.7) | 0.08 | 11 (36)  191 (23) | 1.8 (0.9-3.8) | 0.12 | 5 (16)  21 (3) | 7.3 (2.6-20.9) | <0.001 |
| Quinolones  Yes  No | 39 (5)  811 (95) | 5 (13)  61 (8) | 1.8 (0.7-4.8) | 0.23 | 16 (41)  174 (22) | 2.5 (1.3-4.9) | 0.004 | 9 (23)  115 (14) | 1.8 (0.8-3.9) | 0.12 | 16 (41)  184 (23) | 2.4 (1.2-4.6) | 0.01 | 5 (13)  20 (3) | 5.8 (2.1-16.4) | <0.001 |
| MDR | 31 (4)  819 (96) | 4 (13)  62 (8) | 1.8 (0.6-5.3) | 0.28 | 14 (45)  176 (22) | 3.0 (1.5-6.2) | 0.002 | 7 (23)  119 (15) | 1.7 (0.7-4.1) | 0.22 | 12 (39)  189 (23) | 2.1 (1.0-4.4) | 0.04 | 4 (13)  21 (3) | 5.6 (1.8-17.5) | 0.001 |

PIP-TAZ, Piperacillin-tazobactam. MDR, multidrug-resistant.
