# Supplementary material for: Acquisition of Pseudomonas aeruginosa and its resistance phenotypes in critically ill medical patients: role of colonization pressure and antibiotic exposure
Source: Crit Care. 2015 May 4;19(1):218. doi: 10.1186/s13054-015-0916-7 (PMC4432505; doi:10.1186/s13054-015-0916-7)
Supplement: Additional file 4: — Multivariate analysis of factors associated with acquisition of resistance to each antipseudomonal agent and MDR. [file 13054_2015_916_MOESM4_ESM.docx]

Additional file 4. Multivariate analysis of factors associated with acquisition of resistance to each antipseudomonal agent and MDR.

| **Variable** | **OR (95% CI)** | **p** |
| --- | --- | --- |
| ***Carbapenem resistance*** | | |
| Male sex | 2.7 (1.3-5.8) | 0.009 |
| Cirrhosis | 3.7 (1.3-10.6) | 0.016 |
| Emergency surgery prior to ICU admission | 4.9 (2.4-10.1) | <0.001 |
| Endoscopy | 3.1 (1.5-6.3) | 0.003 |
| Carbapenem >3 days | 2.7 (1.4-5.2) | 0.003 |
| ***Piperacillin-tazobactam resistance*** | | |
| Male sex | 3.8 (1.4-10.2) | 0.009 |
| Emergency surgery prior to ICU admission | 2.7 (1.1-7.0) | 0.037 |
| Endoscopy | 2.8 (1.2-6.6) | 0.015 |
| Nasogastric tube >3days | 4.4 (1.6-12.0) | 0.004 |
| Amikacin >3 days | 5.9 (1.9-18.7) | 0.003 |
| ***Ceftazidime resistance*** | | |
| Male sex | 3.0 (1.3-6.8) | 0.008 |
| Emergency surgery prior to ICU admission | 2.6 (1.2-5.8) | 0.015 |
| Immunosuppressive treatment | 2.8 (1.2-6.3) | 0.015 |
| Intubation >3 days | 4.5 (2.3-9.2) | <0.001 |
| ***Quinolone resistance*** | | |
| Male sex | 2.4 (1.1-5.4) | 0.027 |
| Emergency surgery prior to ICU admission | 3.3 (1.4-7.4) | 0.005 |
| Endoscopy | 3.2 (1.5-6.7) | 0.003 |
| Nasogastric tube >3days | 3.8 (1.6-9.2) | 0.002 |
| Amikacin>3 days | 5.0 (1.6-15.3) | 0.005 |
| ***MDR*** | | |
| Male sex | 3.1 (1.2-7.8) | 0.018 |
| Emergency surgery prior to ICU admission | 2.8 (1.1-7.3) | 0.03 |
| Endoscopy | 3.7 (1.6-8.5) | 0.002 |
| Nasogastric tube >3days | 3.3 (1.3-8.5) | 0.014 |
| Amikacin >3 days | 4.5 (1.3-15.2) | 0.017 |

MDR, multidrug-resistant; ICU, Intensive Care Unit.
